# Discovery of a Virulent Population of *Meloidogyne graminicola* Overcoming the Rice Resistance Gene *MG1* and Population Genetic Analysis Based on *mtCOI* Sequences

**DOI:** 10.3390/plants15193031

**Published:** 2026-10-03

**Authors:** Ao Xu, Borong Lin, Qiuling Huang, Yuqing Cao, Yaya Fan, Zhong Ding, Kan Zhuo

**Affiliations:** 1 College of Plant Protection, South China Agricultural University, Guangzhou 510642, China; xuao010626@163.com (A.X.); boronglin@scau.edu.cn (B.L.); 31000597@scau.edu.cn (Q.H.); caoyuqing22@163.com (Y.C.); 19286649913@163.com (Y.F.); 2 College of Plant Protection, Hunan Agricultural University, Changsha 410128, China; dingzh@hunau.net

**Keywords:** *Meloidogyne graminicola*, virulence, *MG1* resistance gene, population genetics, China

## Abstract

The rice root-knot nematode (*Meloidogyne graminicola*) is a major pathogen of rice. The resistance gene *MG1* in the cultivar Zhonghua 11 (ZH11) confers effective resistance against this pathogen. To date, only one virulent population overcoming ZH11 has been reported, from Cambodia, but whether it overcomes *MG1* remains unknown. Here, we collected and identified 31 *M. graminicola* populations from southern China. Ten of these populations were tested on ZH11. Two of the 10 tested populations were virulent, GZSD1 from Guangdong and HUNSD28 from Hunan, with reproduction factors (Rf) of 3.11 and 1.67, respectively. GZSD1 was also reproduced on NIL-*MG1*, a near-isogenic line developed by introgressing *MG1* into the susceptible variety Huazhan, with an Rf of 2.78. Moreover, GZSD1 showed no significant differences in developmental progression or parasitic ability, either from an avirulent population HAINSD24 on the susceptible cultivar Nipponbare or between the resistant line ZH11 and the susceptible cultivar Nipponbare, suggesting that GZSD1 can overcome *MG1*-mediated resistance without a significant fitness cost under the tested conditions. Population genetic analysis based on *mtCOI* sequences revealed high haplotype diversity and low genetic differentiation with weak geographic structuring among the sampled populations. A signal of population expansion was detected. These findings identify the first virulent *M. graminicola* population overcoming *MG1*-mediated resistance in rice and suggest that virulence may have the potential to spread. This work highlights the threat to *MG1*-based resistance and calls for monitoring and mechanistic studies to support sustainable rice breeding.

## 1. Introduction

Rice (*Oryza sativa* L.) is one of the most important food crops in the world, feeding more than half of the global population [1]. However, its production is limited by various pathogens, among which plant-parasitic nematodes are the major ones. To date, over 200 nematode species have been reported to infect rice, with the root-knot nematode (RKN) *Meloidogyne graminicola* considered one of the most destructive [2]. After invading rice roots, *M. graminicola* usually induces gall formation at root tips, altering the root vascular system. These changes impair root development and function, reduce water and nutrient transport to shoots, and cause stunted growth, leaf chlorosis, early flowering, and, in severe cases, unfilled grains, leading to considerable yield losses [3,4,5].

In recent years, the occurrence of *M. graminicola* in China has expanded, and its damage has become increasingly severe. These trends have been suggested to be associated with global warming and the widespread adoption of direct-seeding rice cultivation [6,7]. Growing resistant varieties is one of the most environmentally friendly, cost-effective, and sustainable approaches to RKN control [5,8]. Considerable progress has been made in identifying resistant sources. Early studies showed that African rice species *O. glaberrima*, *O. longistaminata*, and *O. rufipogon* possess resistance to *M. graminicola* [9,10]. Subsequently, resistant varieties were also identified in Asian cultivated rice (*O. sativa*), such as Khao Pahk Maw from Thailand, LD24 from Sri Lanka, and Zhonghua 11 (ZH11), Shenliangyou 1, Cliangyou 4418 and Huaidao 5 from China [11,12,13], providing key genetic resources for the green control of this nematode.

Classical genetic studies have indicated that rice resistance to *M. graminicola* is controlled by quantitative trait loci (QTLs). For example, Dimkpa et al. [11] identified 11 QTLs associated with gall number using a panel of cultivated rice accessions, including landraces and elite cultivars. Galeng-Lawilao et al. [14] mapped multiple minor-effect QTLs for resistance through phenotypic-genotypic association analysis of a recombinant inbred line population across two seasons. Lahari et al. [15] employed QTL-seq with bulked segregant analysis using two resistant Asian rice varieties, LD24 and Khao Pahk Maw, crossed with the moderately susceptible variety Vialone Nano, and identified a 23 Mb resistance locus on chromosome 11 that was common to both resistant donors. These findings provide a basis for further fine mapping and breeding utilization.

Recently, based on genetic segregation analysis, Nguyen et al. [16] proposed that two complementary dominant genes might contribute to ZH11 resistance; however, the corresponding genetic loci were not mapped, and their molecular identities remained unresolved. Subsequently, Wang et al. [17] cloned and characterized *MG1* from ZH11, which is the first and, to date, the only resistance gene against *M. graminicola* in rice, providing a key molecular resource for breeding. However, Nguyen et al. [18] isolated a virulent population, named Mg.Vir.P1, from Cambodian fields that can overcome the resistance of ZH11, although it remains unknown whether this population can break *MG1*-mediated resistance. More importantly, no *M. graminicola* population capable of breaking ZH11 or *MG1*-mediated resistance has previously been reported in China, and whether such populations exist, and if so, what their genetic background is, remains unclear. Accordingly, we collected rice RKN populations from southern China, identified them using morphological and molecular methods, and evaluated their virulence on ZH11, followed by further assessment of *MG1*-mediated resistance in a representative virulent population. We also analysed *mtCOI* sequence variation to examine the genetic diversity and population structure of the sampled populations and to place the detected virulent populations within this genetic background. Our results provide the first evidence of *M. graminicola* virulent populations in China capable of infecting ZH11, including one population that overcomes *MG1*-mediated resistance, the first such case worldwide. The sampled populations showed high haplotype diversity and low genetic differentiation with weak geographic structuring, suggesting that virulent populations may have the potential to spread. These findings highlight the need for monitoring and diversified resistance strategies in rice breeding.

## 2. Results

### 2.1. Identification of M. graminicola Virulent Populations

#### 2.1.1. Species Identification

**Figure 1:**
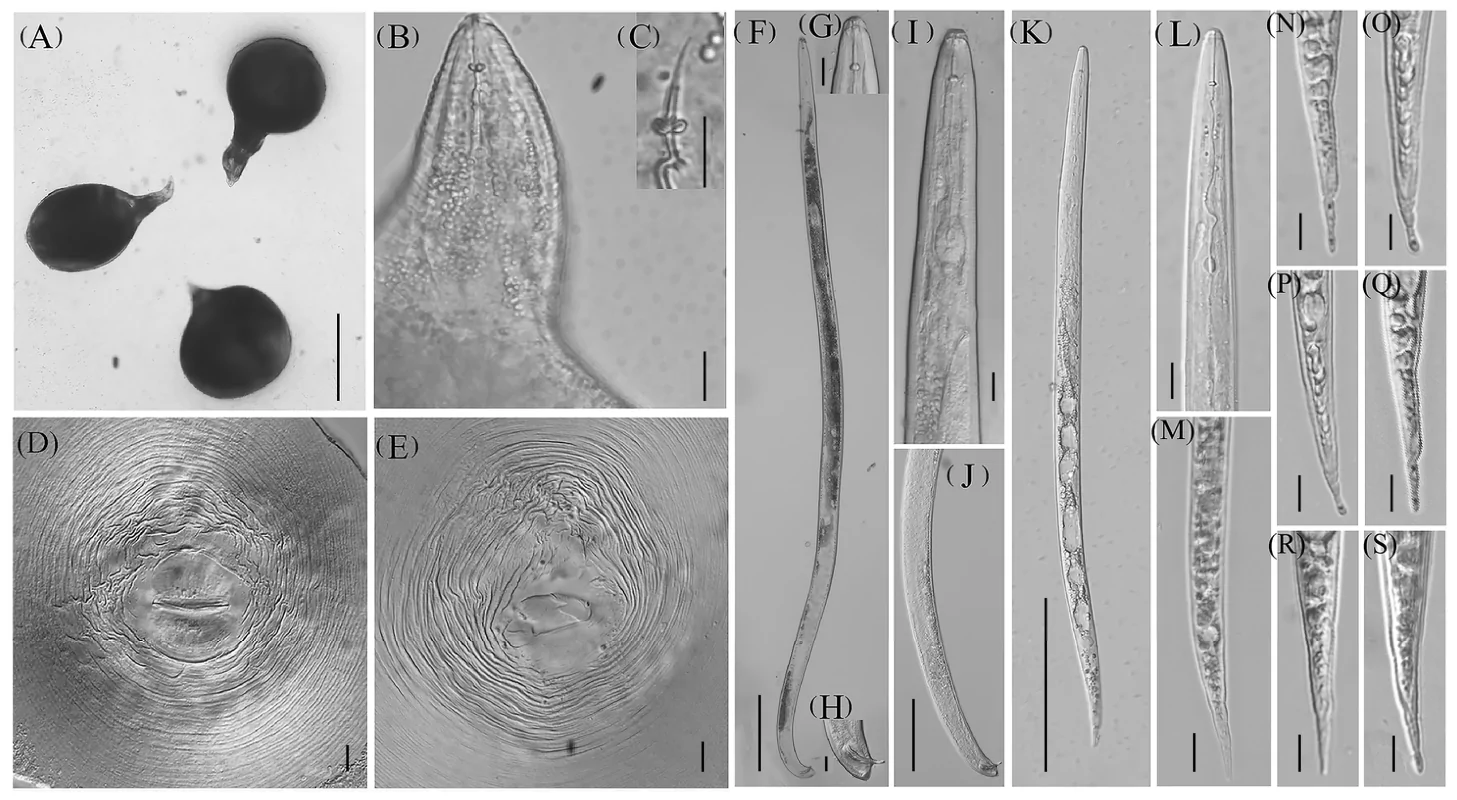
Photomicrographs of the *Meloidogyne graminicola* population GZSD1: (**A**) entire body of female; (**B**) anterior region of female; (**C**) stylet of female; (**D**,**E**) perineal pattern; (**F**) entire body of male; (**G**) lip region and stylet of male; (**H**) spicule and tail of male; (**I**) anterior region of male; (**J**) posterior region of male; (**K**) entire body of second-stage juvenile (J2); (**L**) anterior region of J2; (**M**–**S**) tail of J2. Scale bars: (**A**,**F**) = 500 µm; (**J**,**K**) = 100 µm; (**B**–**E**,**G**–**I**,**L**–**S**) = 10 µm.

Combining traditional morphological taxonomic characters with molecular data, all 31 RKN populations were identified as *Meloidogyne graminicola* (Figure 1; Table 1).

Female: Body swollen, pear-shaped, with a narrow neck; stylet delicate, knobs rounded with anterior margin sloping posteriorly; excretory pore anterior to the median bulb and posterior to the stylet base; perineal pattern oval to round, with smooth striae and low dorsal arch; lateral lines absent.

Male: Vermiform; lip region rounded, high, with distinct annulations; stylet robust, knobs rounded and sloping posteriorly; excretory pore behind the nerve ring; spicules arcuate, gubernaculum rod-shaped; tail short, with bluntly rounded terminus.

Second-stage juvenile (J2): Vermiform, body tapering gradually toward both ends; lip region not offset; stylet slender, knobs small, rounded, sloping posteriorly; excretory pore at or slightly behind the level of the nerve ring; tail slender, hyaline tail region with irregular annulations; tail terminus rounded, often slightly clavate.

These morphological and morphometric characteristics were consistent with the description of *M. graminicola* [19,20].

**Figure 2:**
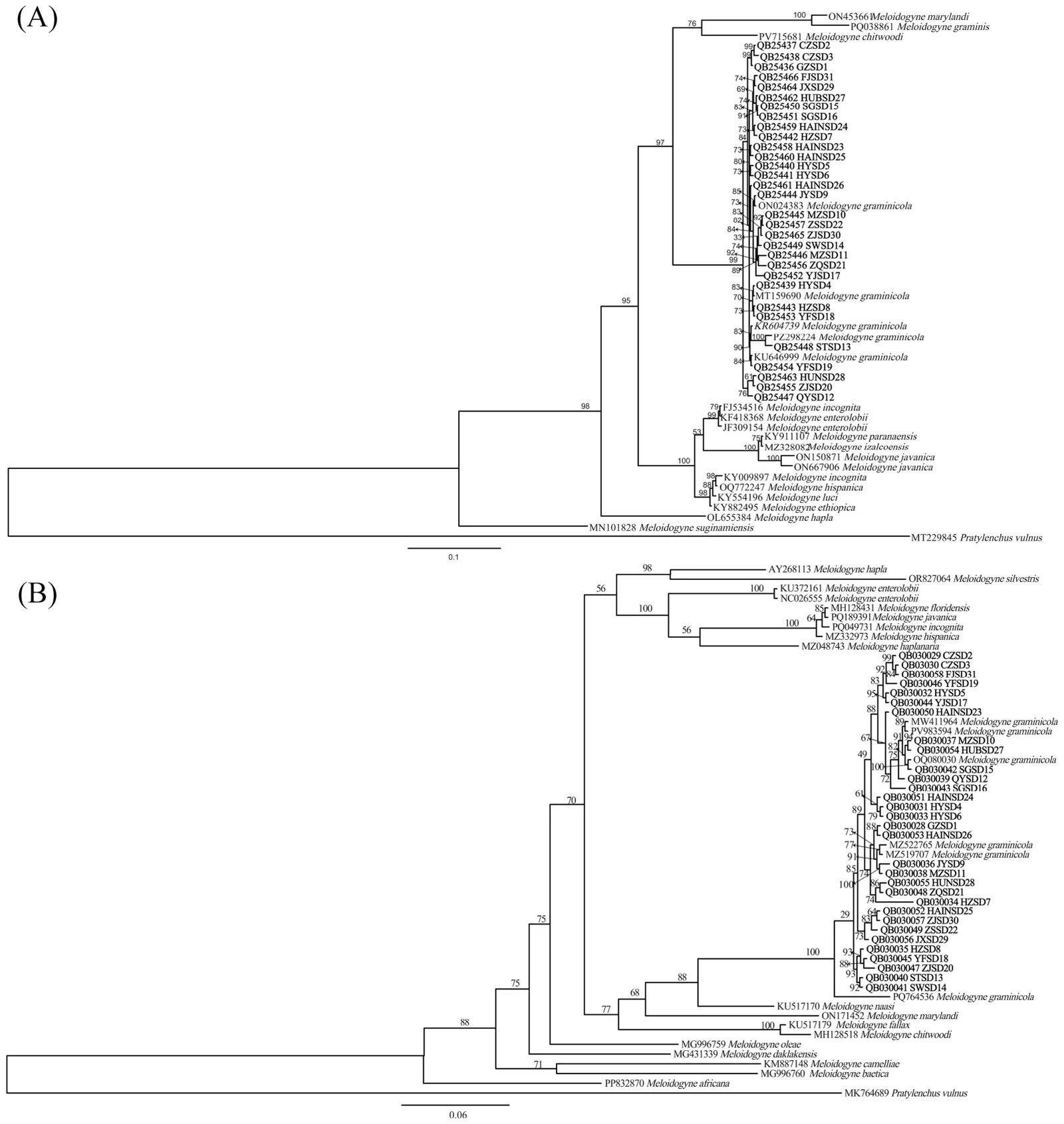
Bayesian phylogenetic trees of *Meloidogyne graminicola*: (**A**) Bayesian phylogenetic tree constructed based on rDNA-ITS sequences; (**B**) Bayesian phylogenetic tree constructed based on mitochondrial *COI* gene sequences. Numbers at branch nodes indicate Bayesian posterior probabilities. Bold font indicates the 31 root-knot nematode populations collected in this study.

Using universal primers (COI F/COI R and TW81/AB28), the mitochondrial *COI* (*mtCOI*) gene and rDNA-ITS region of 31 RKN populations were amplified by PCR and sequenced. The results showed that the *mtCOI* sequences of these populations were 413–418 bp in length, and the rDNA-ITS sequences were 560–578 bp. BLAST searches against the GenBank database revealed that all sequences exhibited the highest similarity to the corresponding sequences of *M. graminicola*, with the *mtCOI* sequences sharing 99.0–100% similarity to the reference *mtCOI* sequence (GenBank accession No. KY250093), and the rDNA-ITS sequences sharing 99.7–100% similarity to the reference rDNA-ITS sequence (GenBank accession No. KR604733). Phylogenetic analysis based on rDNA-ITS and *mtCOI* gene sequences indicated that the 31 nematode populations collected in this study formed a highly supported monophyletic clade (0.99 and 1.00 Posterior probability, respectively) with known populations of *M. graminicola* (Figure 2).

#### 2.1.2. Screening for *M. graminicola* Populations Virulent to Rice Cultivar ZH11

**Figure 3:**
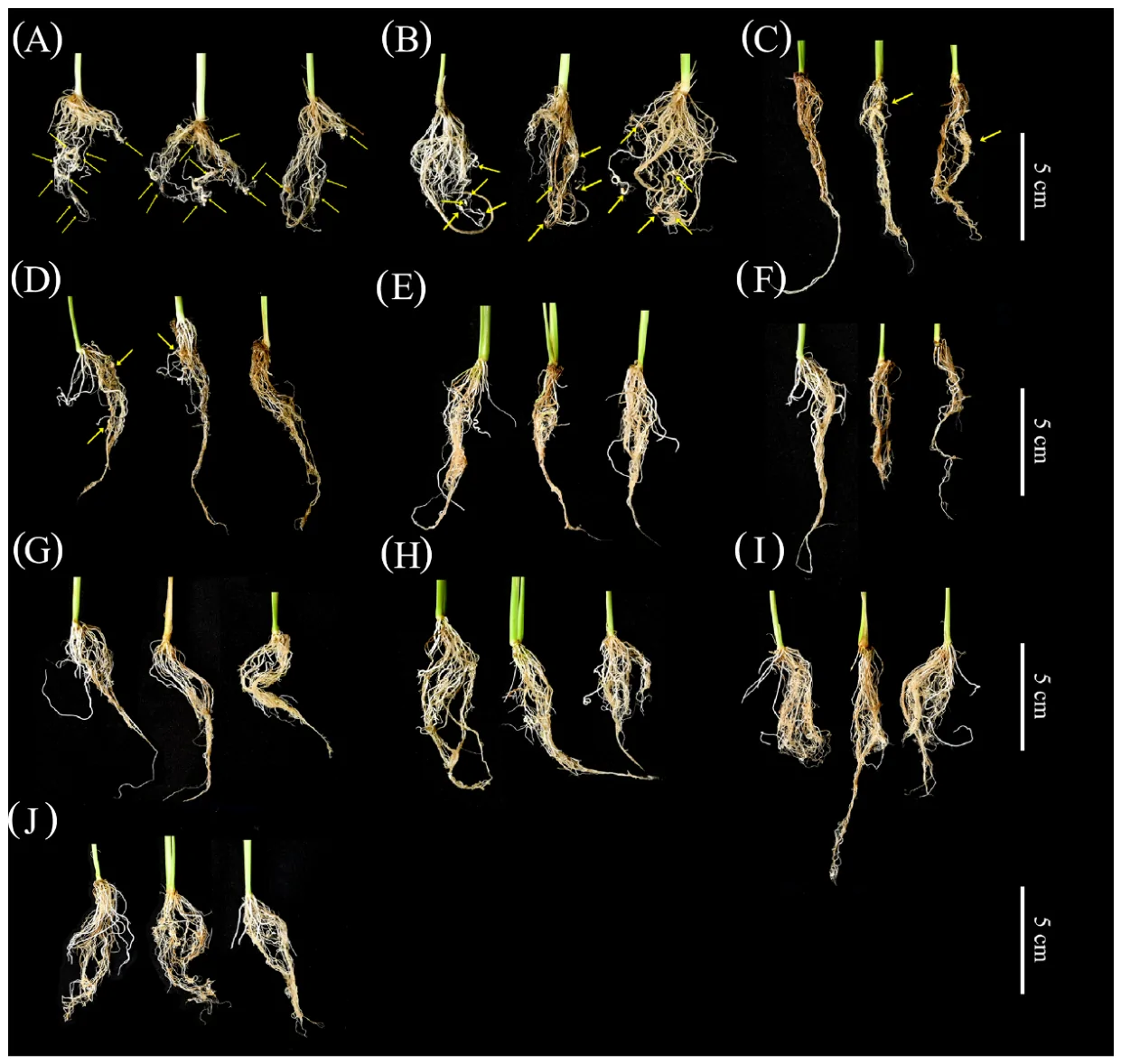
Root symptoms on resistant rice cultivar Zhonghua 11 (ZH11) after inoculation with various geographical populations of *Meloidogyne graminicola*. Letters (**A**–**J**) correspond to populations GZSD1, HUNSD28, ZJSD30, MZSD10, HYSD6, QYSD12, SGSD15, HAINSD23, HAINSD24, and HAINSD26, respectively. Yellow arrows indicate root galls.

**Figure 4:**
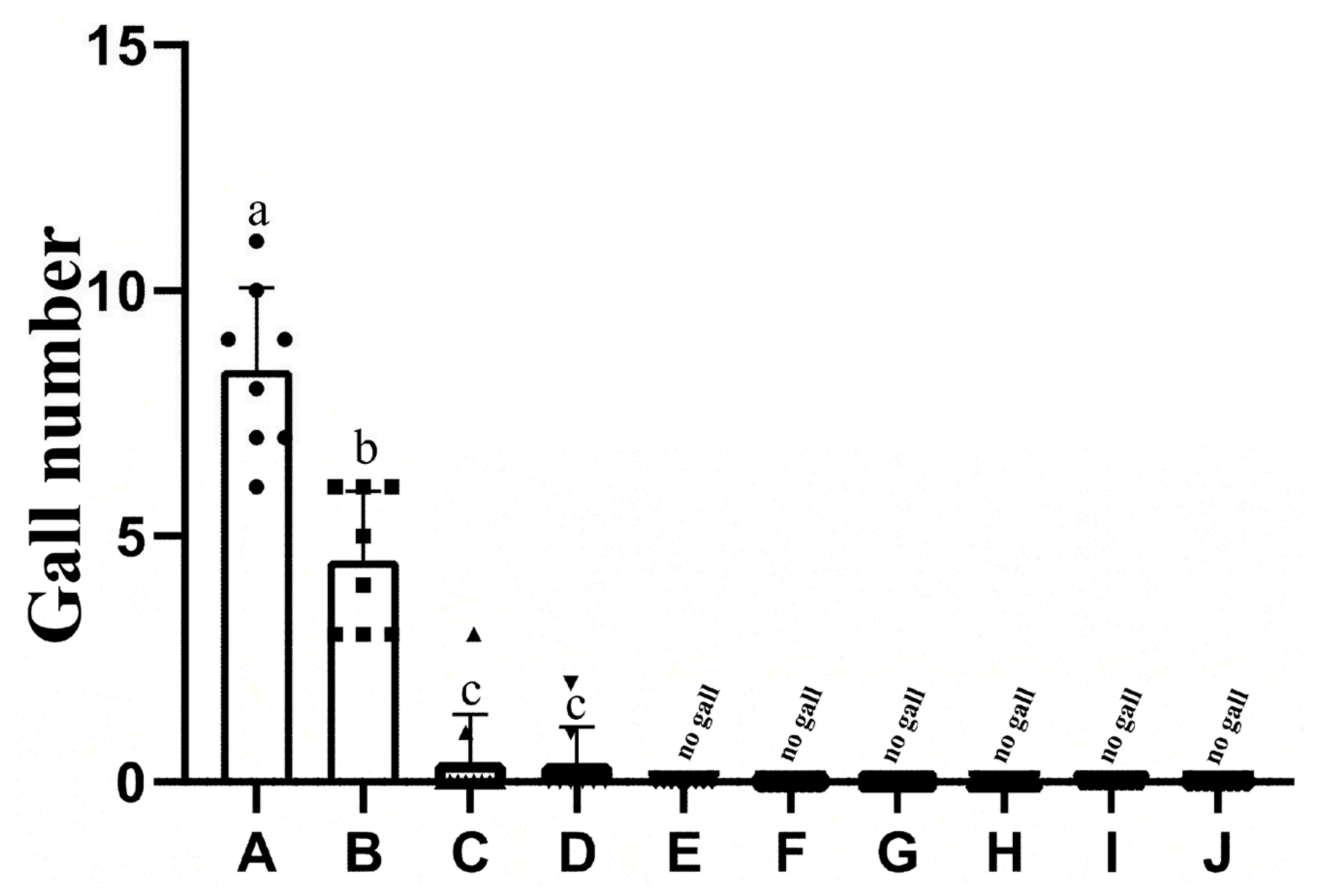
Root-gall numbers on resistant rice cultivar Zhonghua 11 (ZH11) after inoculation with various geographical populations of *Meloidogyne graminicola*. Letters A–J correspond to populations GZSD1, HUNSD28, ZJSD30, MZSD10, HYSD6, QYSD12, SGSD15, HAINSD23, HAINSD24, and HAIND26, respectively. Bars represent means ± SD, and individual symbols represent biological replicates. Different lowercase letters indicate significant differences among the gall-forming populations based on generalized linear models targeted contrasts with Holm adjustment (*p* < 0.05). No root galls were observed in populations E–J.

Ten of the 31 populations were inoculated onto the roots of rice cultivar ZH11. The results showed that two populations (GZSD1 from Guangzhou, Guangdong, and HUNSD28 from Changde, Hunan) successfully infected the roots and completed their life cycle. At 14 days post-inoculation (dpi), the mean gall counts produced by these two populations were 8.38 ± 1.69 and 4.50 ± 1.41, respectively. At 21 dpi, the reproduction factors (Rf) were 3.11 for GZSD1 and 1.67 for HUNSD28. In addition, two other populations, MZSD10 (Meizhou, Guangdong) and ZJSD30 (Ningbo, Zhejiang), formed only a few galls on ZH11, with mean counts of 0.40 ± 0.97 and 0.38 ± 0.74, respectively, and no egg masses were observed. No gall formation was observed for the remaining tested populations (Figure 3 and Figure 4). These results indicate that the two populations from Guangzhou and Changde are virulent populations capable of overcoming the resistance of rice cultivar ZH11.

#### 2.1.3. GZSD1 Population Overcomes the Major Resistance Gene *MG1* in Rice

**Figure 5:**
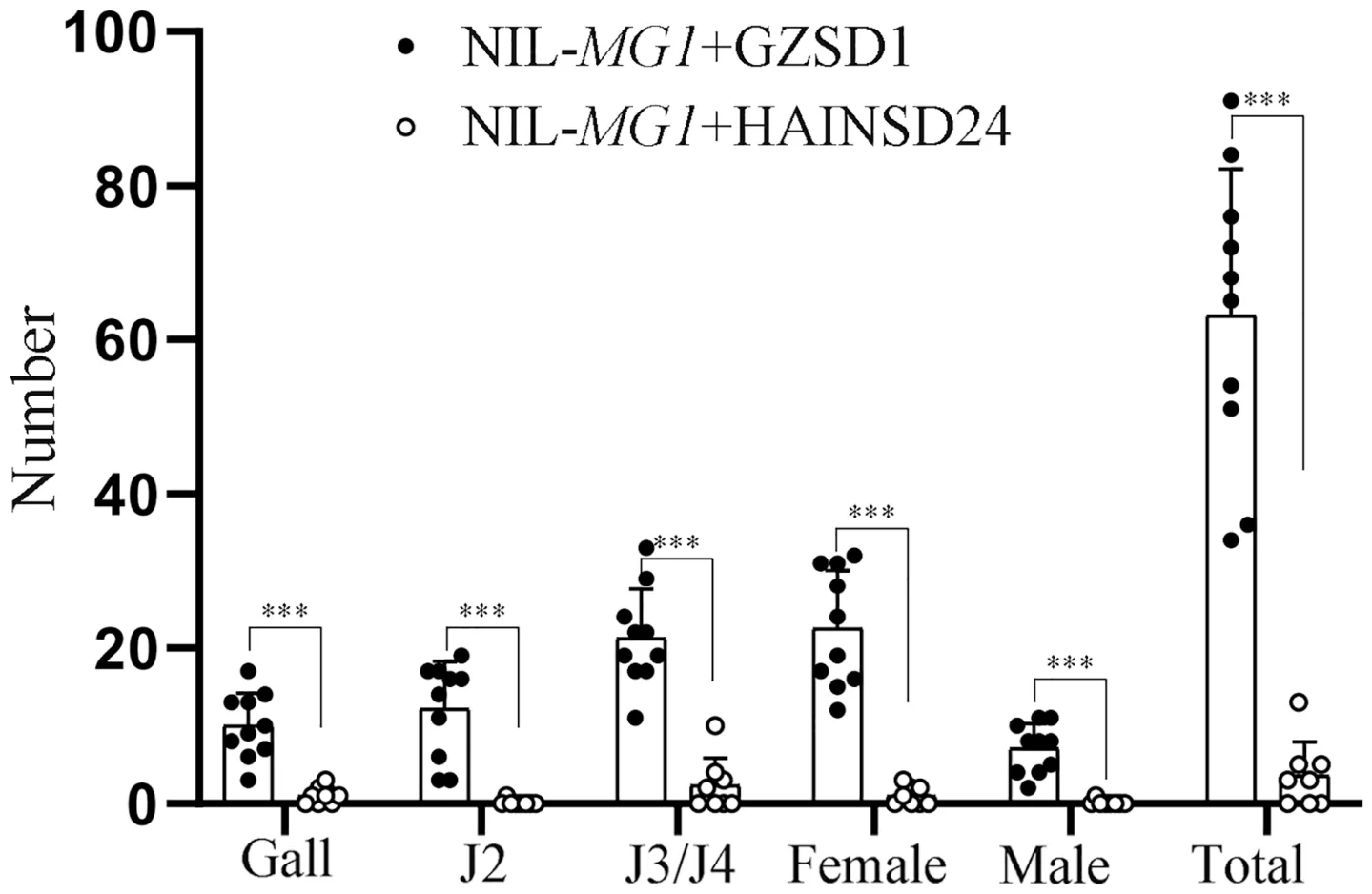
Comparative parasitism of virulent (GZSD1) and avirulent (HAINSD24) *Meloidogyne graminicola* populations on a near-isogenic line NIL-*MG1*, which was developed by introgressing *MG1* locus into the susceptible cultivar Huazhan. J2: second-stage juveniles; J3/J4: third- and fourth-stage juveniles. Bars represent means ± SD, and individual dots represent biological replicates. Count data were analyzed using generalized linear models. Asterisks indicate significant differences between populations (***, adjusted *p* < 0.001).

To further test whether the virulent populations could overcome the major resistance gene *MG1*, GZSD1 was inoculated onto the roots of NIL-*MG1* (a near-isogenic line developed by introgressing the *MG1* locus into the susceptible variety Huazhan). At 14 dpi, GZSD1 produced an average of 10.0 ± 4.24 galls per plant, indicating strong infectivity, whereas the avirulent control HAINSD24 produced significantly fewer galls and also contained significantly fewer nematodes inside the roots (Figure 5). At 21 dpi, the Rf of GZSD1 on NIL-*MG1* reached 2.78, whereas no egg masses were observed in HAINSD24-inoculated plants. Collectively, these results suggest that the Guangzhou-derived population GZSD1 is capable of overcoming *MG1*-mediated resistance under the tested conditions.

### 2.2. Performance of Virulent Population GZSD1 Relative to an Avirulent Population on a Susceptible Cultivar and on Resistant and Susceptible Rice Cultivars

**Figure 6:**
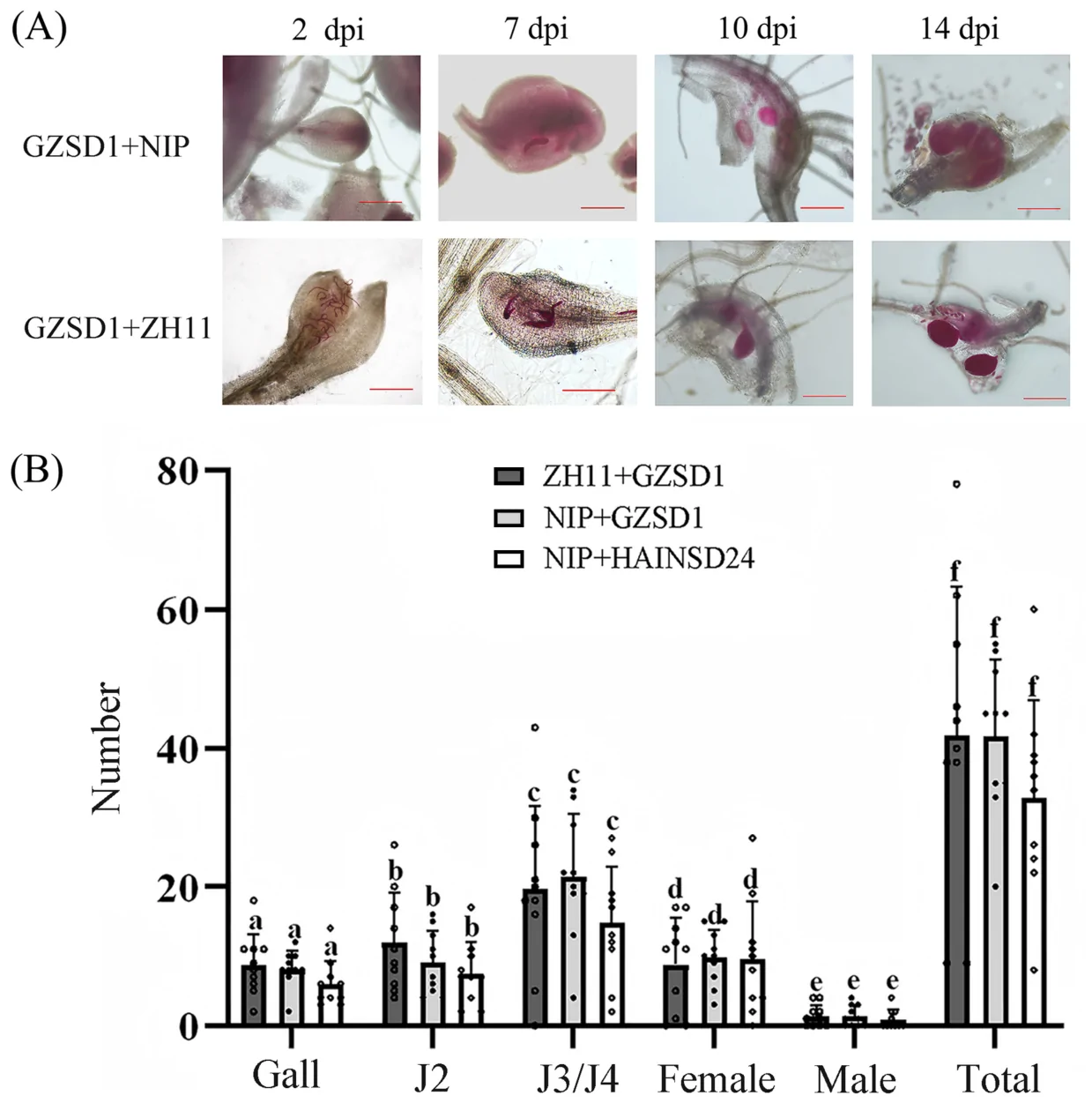
Development and parasitic ability of the virulent *Meloidogyne graminicola* population GZSD1. (**A**) Comparison of GZSD1 development in roots of susceptible (Nip) and resistant (ZH11) rice cultivars. (**B**) Comparison of GZSD1 parasitism on ZH11 and Nip with that of the avirulent population HAINSD24 on Nip. Nip: *Oryza sativa* cv. Nipponbare; ZH11: *O. sativa* cv. Zhonghua 11; J2: second-stage juveniles; J3/J4: third- and fourth-stage juveniles. Bars represent means ± SD, and individual symbols represent biological replicates. Count data were analyzed using generalized linear models. Bars sharing the same lowercase letter are not significantly different among the three treatments (*p* > 0.05). Scale bars: 500 µm.

To compare the parasitic ability of the virulent population GZSD1 with that of the avirulent population HAINSD24 on the susceptible cultivar Nipponbare and to compare the parasitic ability of GZSD1 between the resistant cultivar ZH11 and Nipponbare, GZSD1 was inoculated onto ZH11 and Nipponbare, and HAINSD24 onto Nipponbare. GZSD1 completed its life cycle on both cultivars by 14 dpi (Figure 6A), and HAINSD24 completed its life cycle on Nipponbare, with no obvious difference in developmental progression. At 2 dpi, slight swelling was observed at the root tips, and numerous vermiform J2s were visible inside the roots. At 7 dpi, the juveniles enlarged and molted, with the tail gradually degenerating, and entered the third- or fourth-stage juvenile phase. At 10 dpi, the juveniles continued to enlarge and female adults began to appear. By 14 dpi, mature egg-laying females were observed within the roots. In addition, GZSD1 did not differ significantly from HAINSD24 on Nipponbare, nor did it differ significantly between ZH11 and Nipponbare, in gall number or nematode number (Figure 6B). These results indicate that GZSD1 from Guangzhou shows no significant difference in parasitic ability from the avirulent population HAINSD24 on the susceptible cultivar Nipponbare and no significant difference between resistant and susceptible rice cultivars, suggesting that no significant fitness cost was detected under the tested conditions.

### 2.3. Population Genetics of M. graminicola Inferred from mtCOI Gene Sequences

#### 2.3.1. Sequence Characteristics of the *mtCOI* Gene in *M. graminicola*

Alignment of the 31 *mtCOI* gene sequences from *M. graminicola* showed sequence similarity ranging from 99.1% to 100%. The average nucleotide contents were A: 28.2%, T: 45.3%, C: 8.4%, and G: 17.9%, with an A+T content of 73.6%, indicating a strong A/T bias. Multiple sequence alignment and analysis using DnaSP v6 detected 10 polymorphic sites (Table 2), which accounted for 2.5% of the total bases. Of these, six were singleton variable sites and four were parsimony-informative sites, comprising 60% and 40% of the polymorphic sites, respectively.

#### 2.3.2. Genetic Diversity, Haplotype Distribution, and Demographic History of *M. graminicola*

**Figure 7:**
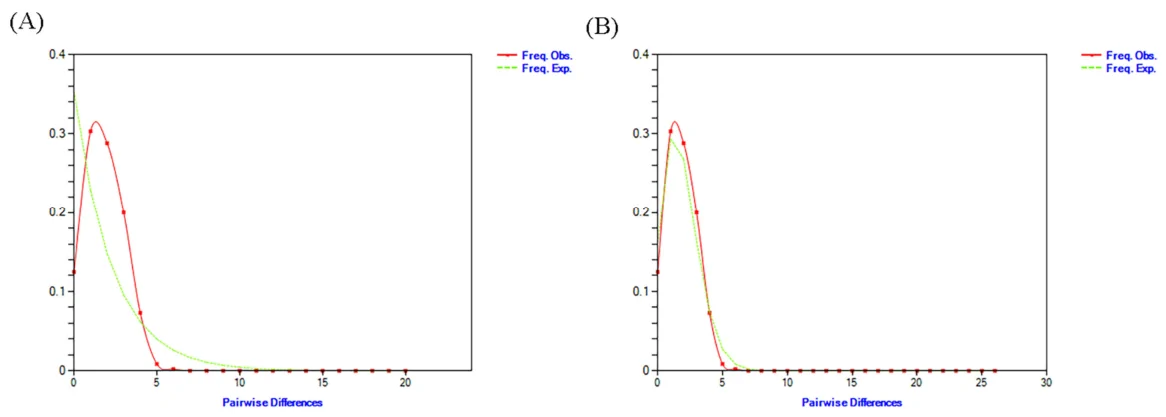
Mismatch distribution analysis of the total *Meloidogyne graminicola* population based on *mtCOI* sequences. (**A**) Mismatch distribution under the constant population size model. The *X*-axis represents the number of pairwise nucleotide differences, and the *Y*-axis represents the observed frequency. Under the constant population size model, the expected curve (green dashed line) exhibits an “L”-shaped declining trend, which clearly deviates from the observed unimodal curve (red solid line), indicating that the population has not been in a long-term stable equilibrium historically. (**B**) Mismatch distribution under the population growth/decline model. The expected curve closely coincides with the observed curve. This result was supported by statistical tests based on coalescent simulations (r = 0.0602, *p* > 0.05; R_2_ = 0.0897, *p* > 0.05).

Genetic diversity indices for the total *M. graminicola* population are summarized in Table 3. The overall haplotype diversity was high (Hd = 0.875), whereas nucleotide diversity was low (π = 0.00456). Among the three climatic zones (Table S1), the North Subtropical population had the highest haplotype diversity (Hd = 1), while the South Subtropical population showed the highest nucleotide diversity (π = 0.00474). The Tropical population exhibited the lowest values for both indices (Hd = 0.7, π = 0.00346). This distribution suggests that the North and South Subtropical populations harbor higher genetic diversity. The mismatch distribution for the total *M. graminicola* population displayed a characteristic unimodal curve that was consistent with the expected sudden expansion model (Figure 7). The Harpending’s raggedness index was low (r = 0.060, *p* > 0.05), and the Ramos-Onsins and Rozas statistic was R_2_ = 0.090 (*p* > 0.05). Neutrality tests (Table 3) yielded negative Tajima’s D (−0.848) and Fu’s Fs (−5.937), but these values were not statistically significant (*p* > 0.05). These results are therefore only suggestive of a possible expansion signal.

Haplotype analysis (Table 4) revealed 12 haplotypes in total. Hap 1 and Hap 3 were the most frequent, with frequencies of 25.81% and 22.58%, respectively. Hap 1 was widely distributed across the South Subtropical and Tropical populations. Three secondary haplotypes (Hap 2, Hap 4, and Hap 10) each occurred at 9.68%, and the remaining seven haplotypes (e.g., Hap 5–7) were found only in single populations (frequency 3.23% each). Overall, the haplotype distribution was characterized by a few relatively common haplotypes and several low-frequency haplotypes.

**Figure 8:**
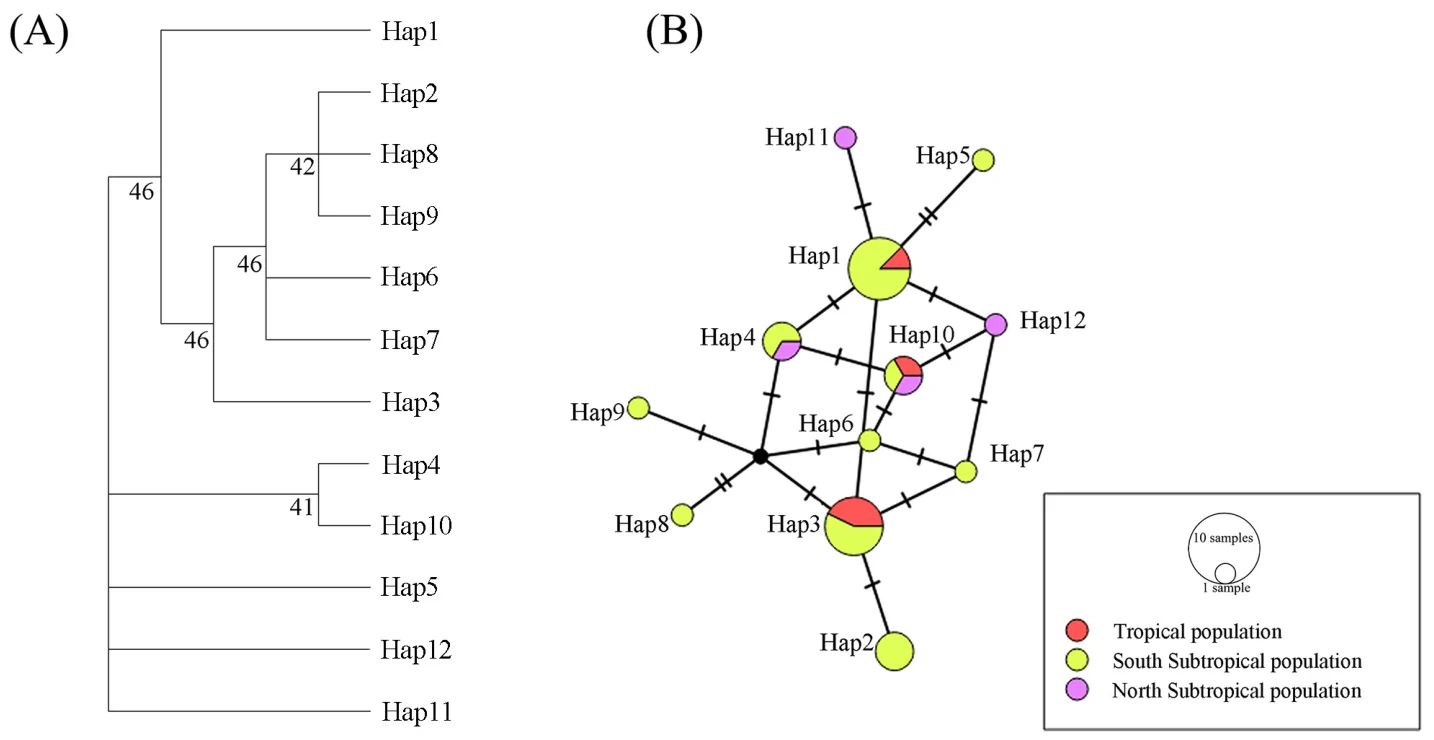
Phylogenetic relationship and haplotype network based on 12 *mtCOI* haplotypes of *Meloidogyne graminicola* populations. (**A**) Maximum parsimony tree constructed using the 12 haplotypes, with bootstrap support values indicated at the nodes. (**B**) Haplotype network. Circle sizes are proportional to the observed frequency of each haplotype, and different colors correspond to the three climatic populations. Each line connecting the haplotypes refers to a mutational step, while black dots represent inferred unsampled intermediate haplotypes.

The phylogenetic tree based on the 12 haplotypes (Figure 8A) exhibited a shallow branching structure, and no geographic clustering was evident in the tree. The haplotype network (Figure 8B) showed a typical star-like topology. Hap 1 and Hap 3 occupied the central positions, from which many secondary and rare haplotypes radiated. Several haplotypes were unique to the North Subtropical population, while haplotypes from different climatic zones were distributed across different parts of the network. Overall, within the resolution of the *mtCOI* fragment analyzed here, the haplotypes showed limited sequence divergence and no clear geographic structuring among the sampled populations. Notably, the two virulent populations identified in this study (GZSD1 and HUNSD28) belonged to Hap 1 and Hap 4, respectively, and occupied different positions in both the tree and the network.

#### 2.3.3. Genetic Differentiation and Population Structure of *M. graminicola*

Genetic differentiation among the three geographic populations were assessed using pairwise fixation indices (F_ST_) (Table 5), which indicated an overall lack of detectable genetic divergence. The F_ST_ value between the Tropical and South Subtropical populations was slightly negative (F_ST_ = −0.107, *p* = 0.976); this value was treated as zero and thus indicated no detectable genetic differentiation between these two groups. Similarly, the F_ST_ values between the North Subtropical population and both the Tropical and South Subtropical populations were not significant. These results indicate weak geographic structuring among the sampled populations at the mitochondrial level.

#### 2.3.4. AMOVA of *M. graminicola* Populations

An analysis of molecular variance (AMOVA) was performed to partition genetic variation among the three geographic groups. The results (Table 6) showed that only 0.61% of the total variation came from among-population differences, while 99.39% resided within populations (*p* < 0.001). This pattern is consistent with the low F_ST_ values observed among populations, suggesting that *M. graminicola* populations from different climatic zones lack significant genetic structure, and most haplotype diversity is attributable to within-population variation.

#### 2.3.5. Mantel Test for Isolation by Distance in *M. graminicola*

**Figure 9:**
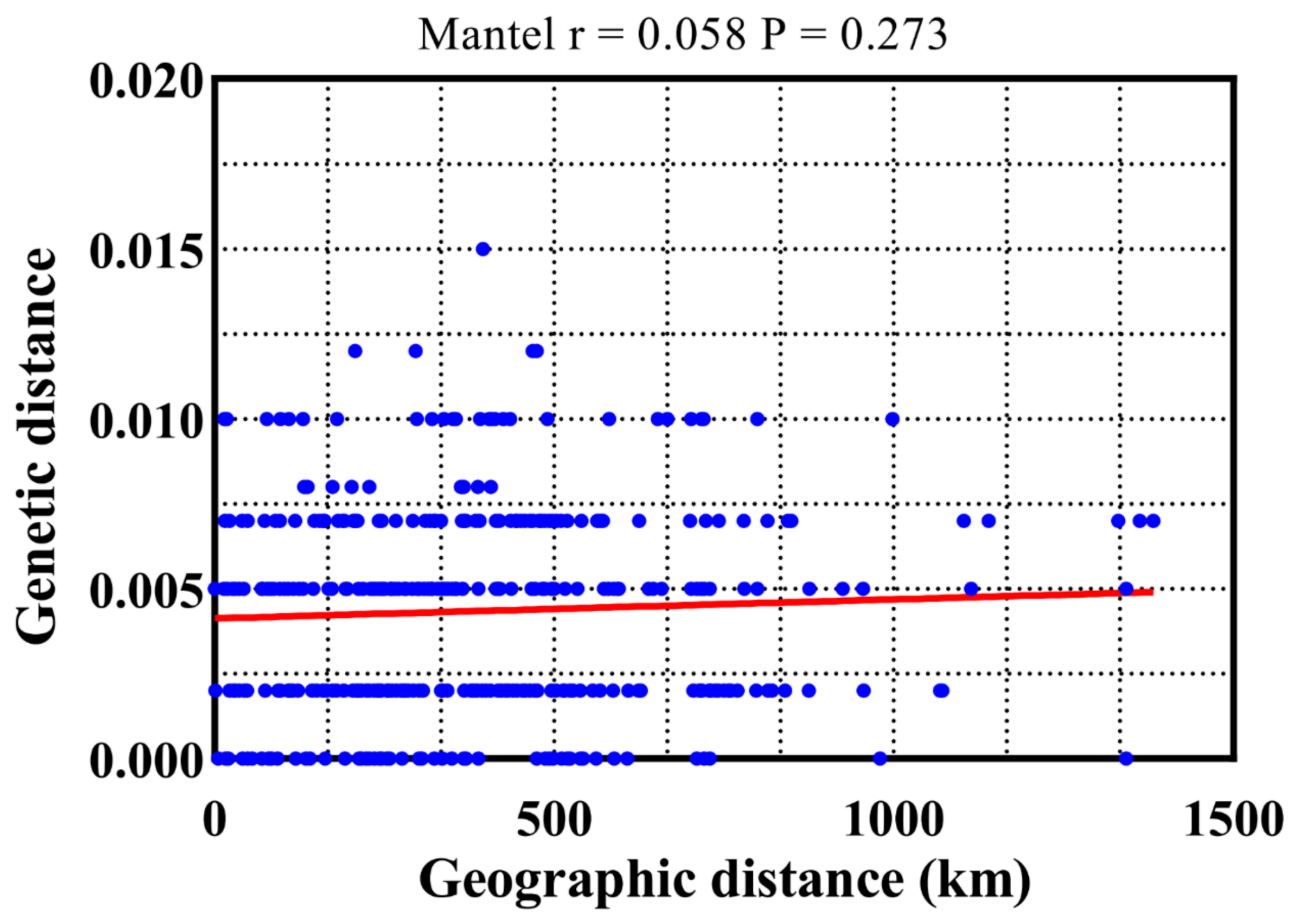
Mantel test for isolation by distance among *Meloidogyne graminicola* populations. The scatter plot displays pairwise genetic distances derived from *mtCOI* sequences against geographic distances (km), with a red solid line indicating the linear regression trend. The Mantel correlation coefficient (*r* = 0.058) and associated *p*-value (*p* = 0.273) reveal no significant positive correlation.

A Mantel test with 999 permutations was performed to examine the relationship between genetic and geographic distances among populations (Figure 9). No significant positive correlation was found (Mantel *r* = 0.058, *p* = 0.273). The flat trend line further indicates that the isolation-by-distance (IBD) model does not adequately explain the observed differentiation pattern. Thus, at the current sampling scale, geographic distance is unlikely to be the main driver of population differentiation in this species.

## 3. Discussion

In this study, we collected and identified 31 *M. graminicola* populations from rice fields in southern China and tested 10 of them for infectivity on the resistant variety ZH11. Importantly, two of the 10 tested populations (GZSD1 and HUNSD28) were able to infect ZH11 and successfully reproduce on it. According to the gene-for-gene hypothesis [21], continuous cultivation of varieties carrying a single resistance gene imposes strong selection pressure on nematode populations, allowing rare virulent variants to reproduce and accumulate over generations [22]. Virulent populations are generally defined as those able to overcome a specific resistance gene and reproduce on that host [23]. However, virulence can also arise without prior exposure to the resistance gene, as reported in other *Meloidogyne* species [24,25]. In *M. graminicola*, virulence studies remain limited. Recently, Nguyen et al. [18] described a field population from Cambodia that could break the resistance of rice cultivar ZH11. Here, we report for the first time that virulent *M. graminicola* populations infect ZH11 in China, suggesting that virulence against this resistant rice variety is not restricted to Cambodia.

Since ZH11 carries *MG1*, the only known resistance gene against this nematode [17], we tested whether our virulent populations could overcome this gene. We inoculated GZSD1 onto the roots of NIL-*MG1*, a near-isogenic line developed by introgressing the *MG1* locus into the susceptible variety Huazhan background [17]. The resistance of NIL-*MG1* to *M. graminicola* was previously validated by comparative inoculation with the susceptible Huazhan, demonstrating that the introgressed *MG1* locus confers resistance in this genetic background [17]. In the present study, the Rf of GZSD1 in NIL-*MG1* was 2.78, whereas no egg masses were observed in HAINSD24-inoculated plants. Although this earlier validation did not include GZSD1 or HAINSD24 and therefore cannot fully replace a direct side-by-side comparison on both NIL-*MG1* and Huazhan, GZSD1 was able to parasitize and reproduce on this functionally validated *MG1*-carrying near-isogenic line, whereas HAINSD24 showed significantly lower parasitic ability and failed to reproduce. The current data and the published validation of NIL-*MG1* suggest that GZSD1 may be able to overcome *MG1*-mediated resistance under the tested conditions. This is the first evidence from a field-collected population of virulence overcoming *MG1*. This finding raises the possibility that *MG1* could be overcome if deployed alone, and since it is currently the only resistance gene available, acquiring additional genes should be a priority in rice breeding.

Notably, GZSD1 completed its life cycle by 14 dpi on both the resistant (ZH11) and susceptible (Nipponbare) varieties, and showed no significant differences from the avirulent population HAINSD24 on Nipponbare, or between ZH11 and Nipponbare, in developmental timing, gall number, or nematode number inside roots. These observations suggest that the virulence of GZSD1 is not associated with a significant fitness cost on the susceptible host under the tested conditions. This finding is noteworthy because classical theory says that mutations or deletions in avirulence effectors that enable resistance breaking typically impose a fitness cost, manifested as reduced infectivity, fewer offspring production, or slower development on susceptible plants [26,27]. Such a fitness cost has also recently been demonstrated in RKNs. Blundell et al. showed that the *Mi-1*-virulent, resistance-breaking *M. javanica* strain VW5 produced significantly fewer eggs than the closely related avirulent strain VW4 on susceptible tomato, cucumber, and rice [28]. However, fitness costs associated with virulence are not universal. For instance, a study on *Puccinia striiformis*, the causal agent of wheat stripe rust, found that loss of *Yr2* avirulence did not affect pathogen fitness [29]. Similarly, Petrillo and Roberts found that although some virulent lines of *M. incognita* capable of overcoming the cowpea resistance gene *Rk* showed reduced egg mass production and fecundity on susceptible cowpea, other virulent lines from the same isolates did not show reduced reproductive ability on susceptible cowpea over 27 generations [30]. As discussed in reference [26], the presence and magnitude of fitness costs can be gene-specific, and pathogens may also acquire compensatory mutations that mitigate such costs. In the present study, no significant fitness cost was detected in GZSD1 under the tested conditions, suggesting that this population may either incur little or no such cost or possess compensatory mechanisms, although further confirmation is needed.

We further performed a population genetic analysis of 31 *M. graminicola* isolates using the *mtCOI* sequence. The populations in southern China showed a distribution pattern characterized by a few relatively common haplotypes and several low-frequency haplotypes, with high haplotype diversity and low nucleotide diversity. The star-like topology of the haplotype network, neutrality test results and mismatch distribution analysis suggested population expansion, consistent with previously reported demographic expansion in this species [6]. In addition, the low genetic differentiation revealed by F_ST_ and AMOVA, together with the lack of a significant isolation-by-distance pattern, indicated relatively weak geographic structuring among the sampled populations. Notably, the two virulent populations (GZSD1 and HUNSD28) were placed separately in the haplotype network and phylogenetic tree. They belonged to Hap 1 and Hap 4, respectively. These results are consistent with the possibility that virulence differentiation in *M. graminicola* is not closely associated with mitochondrial lineage evolution. A similar finding was reported by Semblat et al. [31], who used AFLP markers to assess genetic diversity in parthenogenetic root-knot nematodes overcoming the tomato *Mi* resistance gene, and found no significant correlation between virulence phenotypes and neutral genome-wide polymorphisms. The underlying reason for this discrepancy lies in the different evolutionary dynamics and timescales reflected by the two marker types. The *mtCOI* gene is maternally inherited, and its mutations are driven primarily by genetic drift, thus reflecting the long-term evolutionary history and broad dispersal routes of populations [32]. Virulence, in contrast, is subject to strong selection and represents a recent micro-evolutionary event, which cannot be captured by slowly evolving, independently inherited mitochondrial sequences.

Together, our results suggest that virulent populations capable of overcoming the *MG1*-mediated resistance without a significant fitness costs under the tested conditions may have already emerged in the field. This finding suggests that resistance breeding should not rely solely on major resistance genes. Instead, new sources of resistance with different mechanisms should be identified, and gene pyramiding strategies should be used to slow the evolution and spread of virulent populations. The population genetic patterns observed in this study are consistent with historical population expansion [6] and weak geographic genetic structuring in *M. graminicola*. Together with possible human-mediated and water-mediated dispersal [33,34], these features may facilitate the spread of virulent populations across rice-growing regions, potentially threatening rice production. However, these potential dispersal pathways require further investigation. In cultivation management, integrated measures such as crop rotation, flooding irrigation, and biological control should be encouraged to reduce the initial inoculum in fields and limit population growth [5].

## 4. Materials and Methods

### 4.1. Nematode Materials

A total of 31 rice RKN samples were collected from rice fields in southern provinces of China between 2023 and 2026 (Table S1). Females and males were dissected from galls. J2s were collected from hatched eggs, following the procedure described by Huang et al. [35].

### 4.2. Morphological Identification

Males and J2s were relaxed with gentle heat, fixed in 4% formaldehyde with 1% glycerin, and processed by the glycerin-ethanol method [36]. Perineal patterns of mature females were prepared following the method described by Hartman and Sasser [37], trimmed, and mounted in glycerin for observation. Measurements and photographs were taken with a Nikon ECLIPSE Ni microscope equipped with a Nikon Digital Sight Camera and NIS-Elements BR 4.13.05 software (Nikon, Tokyo, Japan).

### 4.3. DNA Extraction, Amplification and Sequencing

Nematode DNA was extracted using the protocol of Subbotin et al. [38]. The rDNA-ITS region and the *mtCOI* gene fragment were amplified using the universal primers TW81/AB28 (5′-GTTTCCGTAGGTGAACCTGC-3′/5′-ATATGCTTAAGTTCAGCGGGT-3′) [39] and COI F/COI R (5′-GATTTTTTGGKCATCCWGARG-3′/5′-CWACATAATAAGTATCATG-3′) [40], respectively. PCR amplification for the rDNA-ITS and *mtCOI* fragments followed the protocols described by Tanha Maafi et al. [41] and He et al. [40], respectively. DNA sequencing was carried out following the method described by Zhuo et al. [42]. All sequences generated in this study were deposited in GenBank, and the corresponding accession numbers are provided in Table S1.

### 4.4. Phylogenetic Analyses

The obtained sequences were compared with nematode sequences in GenBank using the BLAST homology search program (blastn, version 2.17.0+; [https://blast.ncbi.nlm.nih.gov/Blast.cgi] accessed on 29 September 2026). The most similar sequences were selected for phylogenetic analyses. Out-group taxa for each dataset were chosen based on previous molecular phylogenetic studies of RKNs [43,44,45]. Multiple sequence alignment was performed using PhyloSuite v1.2.3, and the aligned sequences were trimmed using Gblocks v0.91b. The optimal nucleotide substitution model for each dataset was selected using ModelFinder v2.2.0 based on the Akaike information criterion (AIC). Bayesian inference was conducted using MrBayes v3.2.7a to construct phylogenetic trees, with Markov chain Monte Carlo (MCMC) runs used to estimate posterior probabilities of the clades. The resulting trees were visualized and edited using TreeView v1.4.0 [46,47,48,49].

### 4.5. Screening of M. graminicola Populations Overcoming ZH11 Resistance

Seeds of rice cultivar ZH11 were surface-sterilized with 75% ethanol and 5% NaClO and then germinated on B5 medium at 28 °C under a 16 h light/8 h dark photoperiod for 14 days. The resulting seedlings were hydroponically cultured in water for 7 days and then transplanted into sterilized sandy loam soil following Huang et al. [35]. For each geographically distinct population, 8–10 plants were inoculated using J2s of *M. graminicola*. Freshly hatched J2 suspensions were adjusted to 200 J2 mL^−1^, and each plant was inoculated with 1 mL suspension, while control plants received sterile water. At 14 dpi, the number of galls per root system was recorded. At 21 dpi, root galls were excised, gently crushed, and incubated at 28 °C for 5 days to allow the eggs inside to hatch. The total number of hatched J2s was used as Pf, and the reproduction factor was calculated as Rf = Pf/Pi, with Pi = 200 J2s per plant following the description of Oostenbrink [50]. A population was considered virulent to ZH11 if it was able to complete its life cycle and reproduce on ZH11, producing eggs and having an Rf > 1 at 21 dpi [23].

### 4.6. Evaluation of MG1-Mediated Resistance to M. graminicola in Rice

Each plant of the NIL-*MG1* was inoculated with 200 J2s of a *M. graminicola* population virulent to ZH11, while control plants were inoculated with 200 J2s of a population avirulent to ZH11. For each treatment, 10 plants served as biological replicates, and two independent experiments were performed. Gall numbers and Rf were assessed at 14 and 21 dpi, respectively.

### 4.7. Development Observation

The development of a *M. graminicola* population virulent to ZH11 was observed within the roots of resistant rice cultivar ZH11 and susceptible cultivar Nipponbare. Developmental stages were distinguished by body shape as follows: parasitic J2s were vermiform; third-(J3) and fourth-stage juveniles (J4) were swollen, sausage- or flask-shaped with a short tail; and adult females were pear-shaped, according to Huang et al. Huang et al. [35]. Each plant was inoculated with 200 J2s, with 8–10 plants used for each rice cultivar. The experiment was independently performed two times.

### 4.8. mtCOI-Based Population Genetic Analysis of M. graminicola

#### 4.8.1. Sequence Variation and Polymorphism Analysis

The *mtCOI* sequences were aligned using MEGA v11 (Molecular Evolutionary Genetics Analysis version 11; Institute for Genomics and Evolutionary Medicine, Temple University, Philadelphia, PA, USA) [51]. Nucleotide composition and polymorphic sites were analyzed with DnaSP v6 (Universitat de Barcelona, Barcelona, Spain) [52]. For each population, genetic diversity indices were calculated, including the percentage of variable sites, haplotype diversity (Hd), nucleotide diversity (π), and average nucleotide differences (K).

#### 4.8.2. Haplotype Network and Phylogenetic Analysis

Haplotypes were determined with DnaSP v6. A maximum parsimony (MP) tree was constructed in MEGA v11 [51], with branch support evaluated by 1000 bootstrap replicates. The tree was visualized and edited in MEGA v11. A haplotype network was also built using PopART v1.7 (University of Otago, Dunedin, New Zealand) [53] with the statistical parsimony method, which illustrated the evolutionary relationships among haplotypes.

#### 4.8.3. Population Genetic Structure and Demographic History Analysis

Populations were grouped according to climatic zones. Pairwise fixation indices (F_ST_) among geographic populations and their associated *p* values were calculated using Arlequin v3.5.2 (University of Bern, Bern, Switzerland) [54] to assess genetic differentiation among populations. Tajima’s D and Fu’s Fs neutrality tests were performed in DnaSP v6 for each population and for all populations combined. In addition, mismatch distribution analysis was conducted in DnaSP v6 as an additional assessment of population demographic history.

#### 4.8.4. Analysis of Molecular Variance

An analysis of molecular variance (AMOVA) was conducted in Arlequin v3.5.2 to partition genetic variation within and among the predefined geographic groups. Significance was tested with 1000 permutations [55].

#### 4.8.5. Association Analysis Between Genetic Differentiation and Geographic Distance

To test for IBD, the correlation between genetic and geographic distances was examined. Pairwise genetic distances were calculated using Nei’s genetic distance in MEGA v11, and geographic distances were derived from the latitude and longitude of each sampling site. The association between the two distance matrices was evaluated using a Mantel test with 999 permutations. A Mantel test was performed in SPSS v18.0 (SPSS Inc., Chicago, IL, USA) to evaluate the correlation between the two matrices [56].

### 4.9. Statistical Analysis and Graphing

Statistical analyses were performed using IBM SPSS v18.0 [56]. Count data were analyzed using generalized linear models (GLMs) with a log link function and either a Poisson or negative binomial distribution, depending on data dispersion. Population or inoculation treatment was included as a fixed effect, and significance was assessed using Wald chi-square tests. Targeted pairwise contrasts were performed where appropriate, with Holm correction applied for multiple comparisons. Differences were considered significant at *p* < 0.05. Data are presented as means ± SD. Graphing was performed using GraphPad Prism 8.0.2 (GraphPad Software, Inc., San Diego, CA, USA) [57].

## 5. Conclusions

This study reports the first occurrence in China of virulent *M. graminicola* populations on ZH11 and identifies the first population worldwide (GZSD1) that overcomes *MG1* resistance under the tested conditions. These findings confirm that virulent nematode populations are present in rice fields of southern China. Genetic analysis based on *mtCOI* sequences shows high haplotype diversity and weak geographic genetic structuring among the sampled populations, with a signal of population expansion. The two virulent populations belonged to distinct mitochondrial haplotypes. The presence of such populations, together with their genetic features, calls for systematic field monitoring, mechanistic studies on GZSD1, and the careful deployment of resistance genes to sustain effective disease control.

## Figures and Tables

**Table 1:** Morphometrics of *Meloidogyne graminicola* females and second-stage juveniles (J2s) from China compared with those from the literature. All measurements are in µm and in the form: mean ± s.d. (range).

Character	GZSD1	HAINSD24	Soares et al. [19]	Golden & Birchfield [20]
Female (*n*)	15	15	20	20
L	651.2 ± 39.1 (590.8–721.3)	624.3 ± 51.7 (584.8–756.6)	556 (450–660)	573 (445–765)
Maximum body diam.	469.2 ± 47.2 (394.4–632.1)	477.6 ± 32.3 (408.3–553.7)	355 (280–440)	419 (275–520)
Vulva slit to anus	19.1 ± 2.1 (18.1–22.3)	-	18.2 (16–22)	-
DGO	3.7 ± 0.2 (3.3–4.5)	3.4 ± 0.2 (3.0–4.7)	3.6 (3–4)	3.2 (3.0–4.0)
Stylet length	11.3 ± 1.2 (10.6–13.6)	12.4 ± 1.5 (11.2–15.7)	12.2 (11–13)	11.0 (10.5–11.5)
J2 (*n*)	25	25	20	20
L	442.2 ± 42.1 (383.7–589.1)	470.7 ± 47.9 (398.7–549.2)	502 (470–520)	441 (415–484)
a	26.6 ± 3.7 (24.2–32.1)	30.7 ± 2.5 (26.1–34.4)	28.4 (21–38)	24.8 (22.3–27.3)
b	4.5 ± 0.5 (3.8–5.2)	5.0 ± 0.5 (4.3–6.2)	-	3.2 (2.9–4.0)
c	6.7 ± 0.5 (5.0–7.1)	6.1 ± 0.6 (4.9–7.3)	6.7 (5.5–7.6)	6.2 (5.5–6.7)
c′	7.0 ± 0.9 (5.6–8.9)	6.8 ± 0.8 (5.4–8.3)	-	4.6 (4.0–5.0)
Maximum body diam.	13.5 ± 0.7 (12.1–16.7)	15.4 ± 0.9 (13.5–16.6)	18.1 (13–24)	12
Stylet length	11.9 ± 0.9 (9.7–13.2)	12.1 ±1.1 (9.8–14.1)	11.6 (11–13)	11.4 (11.0–12.0)
Tail	72.4 ± 6.7 (62.5–82.6)	77.6 ± 9.9 (63.8–84.2)	73 (66–80)	70.9 (67.0–76.0)
hyaline region	17.5 ± 2.8 (14.2–21.5)	20.5 ± 3.5 (16.1–24.4)	19.3 (16–22)	17.9 (14.0–21.0)
Anterior end to excretory pore	87.2 ± 8.1 (79.1–91.3)	81.2 ± 7.3 (70.1–92.6)	72 (65–80)	-
DGO	2.8 ± 0.5 (1.9–4.2)	2.9 ± 0.7 (1.9–4.5)	2.7 (2–3)	2.8 (2.8–3.4)

Note: L, Body length; DGO, distance of dorsal oesophageal gland orifice to base of stylet; a = L/max. width; b = L/oesophageal length; c = L/tail length; c′ = tail length/anal body width.

**Table 2:** Distribution of variable sites across the *mtCOI* gene of *Meloidogyne graminicola*.

Variant Numbers	S-Singleton Sites	Parsimony-Informative Sites
2	50, 146, 160, 171, 302, 357	11, 17, 20, 61
3	None	None
4	None	None

Note: The number 2, 3, and 4 represent the counts of different nucleotide variants at those sites, whereas the remaining numbers indicate the nucleotide positions of the variable sites in the sequences.

**Table 3:** Genetic diversity and neutrality tests for *Meloidogyne graminicola* populations inferred from *mtCOI* gene sequences.

Group	*n*	Number of Mutations	Haplotype Diversity (Hd)	Nucleotide Diversity (π)	Average Number of Nucleotide Differences (K)	Fu’s Fs	Tajima’s D
Tropical population	5	3	0.7	0.00346	1.4	0.061	−0.175
South subtropical population	22	10	0.866	0.00474	1.887	−4.494	−0.789
North subtropical population	4	4	1	0.00453	1.833	−2.017	1.089
Total	31	12	0.875	0.00456	1.828	−5.937	−0.848

**Table 4:** Haplotype distribution among *Meloidogyne graminicola* populations inferred from *mtCOI* sequences.

Haplotype	Haplotype Sequence	Number	Population Code	Haplotype Frequency
Hap1	GTGTTTGTTT	8	GZSD1, HZSD8, MZSD10, MZSD11, STSD13, SWSD14, YFSD18, HAINSD26	25.81%
Hap2	TTGTCTGTTT	3	CZSD2, CZSD3, FJSD31	9.68%
Hap3	TTGTTTGTTT	7	HYSD4, HYSD5, HYSD6, YJSD17, ZJSD20, HAINSD23, HAINSD24	22.58%
Hap4	GTATTTGTTT	3	HZSD7, ZQSD21, HUNSD28	9.68%
Hap5	GTGTTCATTT	1	JYSD9	3.23%
Hap6	TAATTTGTTT	1	QYSD12	3.23%
Hap7	TAGTTTGTTT	1	SGSD15	3.23%
Hap8	TTATTTGTCA	1	SGSD16	3.23%
Hap9	TTAATTGTTT	1	YFSD19	3.23%
Hap10	GAATTTGTTT	3	ZSSD22, HAINSD25, ZJSD30	9.68%
Hap11	GTGTTTGCTT	1	HUBSD27	3.23%
Hap12	GAGTTTGTTT	1	JXSD29	3.23%

**Table 5:** Pairwise genetic differentiation (F_ST_) and corresponding statistical significance (*p*-values) among *Meloidogyne graminicola* populations based on *mtCOI* sequences.

Population	Tropical Population	South Subtropical Population	North Subtropical Population
Tropical population	-	0.975	0.179
South subtropical population	−0.106	-	0.082
North subtropical population	0.133	0.116	-

Note: Values below the diagonal are pairwise F_ST_ estimates, and values above the diagonal are the corresponding *p*-values calculated via 10,000 permutations. A negative F_ST_ value indicates zero differentiation. None of the pairwise comparisons were statistically significant (*p* > 0.05).

**Table 6:** Analysis of molecular variance (AMOVA) within and among *Meloidogyne graminicola* groups inferred from *mtCOI* gene sequences.

Source of Variation	d.f	Sum of Squares	Variance Components	Percentage of Variation
Among populations	2	5.133	0.014	0.60812
Within populations	28	65.641	2.344	99.39188
Total	30	70.774	2.359	100

## Data Availability

Data are contained within the article and its Supplementary Materials. Further inquiries can be directed to the corresponding author.

## Supplementary Materials

The following supporting information can be downloaded at https://www.mdpi.com/article/10.3390/plants15193031/s1: Table S1: Sampling sites for rice root-knot nematode.
