# Supplementary material for: Discovery of a Virulent Population of Meloidogyne graminicola Overcoming the Rice Resistance Gene MG1 and Population Genetic Analysis Based on mtCOI Sequences
Source: Plants (Basel). 2026 Oct 3;15(19):3031. doi: 10.3390/plants15193031 (PMC13666895; doi:10.3390/plants15193031)
Supplement: Supplementary file 1 [file plants-15-03031-s001.zip › plants-4570979-supplementary.pdf]

Table S1 Sampling sites for rice root-knot nematode

No.	Sample Code	Sampling Site	Sampling year	Longitude	Latitude	ITS	*mtCO1*	Virulence screening
1	GZSD1	Guangzhou, Guangdong	2023	113.65	23.24	QB025436	QB030028	Tested
2	CZSD2	Chaozhou, Guangdong	2023	116.65	23.64	QB025437	QB030029	Not tested
3	CZSD3	Chaozhou, Guangdong	2024	116.69	23.57	QB025438	QB030030	Not tested
4	HYSD4	Heyuan, Guangdong	2024	114.64	23.57	QB025439	QB030031	Not tested
5	HYSD5	Heyuan, Guangdong	2024	115.23	24.08	QB025440	QB030032	Not tested
6	HYSD6	Heyuan, Guangdong	2024	114.67	23.67	QB025441	QB030033	Tested
7	HZSD7	Huizhou, Guangdong	2025	114.49	23.02	QB025442	QB030034	Not tested
8	HZSD8	Huizhou, Guangdong	2025	114.48	23.03	QB025443	QB030035	Not tested
9	JYSD9	Jieyang, Guangdong	2025	116.53	23.52	QB025444	QB030036	Not tested
10	MZSD10	Meizhou, Guangdong	2024	116.52	24.35	QB025445	QB030037	Tested
11	MZSD11	Meizhou, Guangdong	2024	116.64	24.17	QB025446	QB030038	Not tested
12	QYSD12	Qingyuan, Guangdong	2024	112.35	24.78	QB025447	QB030039	Tested
13	STSD13	Shantou, Guangdong	2024	116.79	23.42	QB025448	QB030040	Not tested
14	SWSD14	Shanwei, Guangdong	2025	115.55	22.71	QB025449	QB030041	Not tested
15	SGSD15	Shaoguan, Guangdong	2024	113.7	24.79	QB025450	QB030042	Tested
16	SGSD16	Shaoguan, Guangdong	2024	113.01	24.75	QB025451	QB030043	Not tested
17	YJSD17	Yangjiang, Guangdong	2024	111.96	22.55	QB025452	QB030044	Not tested

No.	Sample Code	Sampling Site	Sampling year	Longitude	Latitude	ITS	*mtCO1*	Virulence screening
18	YFSD18	Yunfu, Guangdong	2024	112.21	22.53	QB025453	QB030045	Not tested
19	YFSD19	Yunfu, Guangdong	2025	112.27	22.85	QB025454	QB030046	Not tested
20	ZJSD20	Zhanjiang, Guangdong	2025	110.3	21.25	QB025455	QB030047	Not tested
21	ZQSD21	Zhaoqing, Guangdong	2024	112.55	23.04	QB025456	QB030048	Not tested
22	ZSSD22	Zhongshan, Guangdong	2024	113.52	22.61	QB025457	QB030049	Not tested
23	HAINSD23	Chengmai, Hainan	2025	110.02	19.74	QB025458	QB030050	Tested
24	HAINSD24	Haikou, Hainan	2025	110.17	20.03	QB025459	QB030051	Tested
25	HAINSD25	Haikou, Hainan	2024	110.37	20.01	QB025460	QB030052	Not tested
26	HAINSD26	Haikou, Hainan	2025	110.37	20.03	QB025461	QB030053	Tested
27	HUBSD27	Wuhan, Hubei	2024	114.36	30.47	QB025462	QB030054	Not tested
28	HUNSD28	Changde, Hunan	2024	113.12	28.15	QB025463	QB030055	Tested
29	JXSD29	Yichun, Jiangxi	2025	114.42	27.77	QB025464	QB030056	Not tested
30	ZJSD30	Ningbo, Zhejiang	2025	121.68	29.87	QB025465	QB030057	Tested
31	ZZSD31	Zhangzhou, Fujian	2025	117.4	23.77	QB025466	QB030058	Not tested
